# Single PA mutation as a high yield determinant of avian influenza vaccines

**DOI:** 10.1038/srep40675

**Published:** 2017-01-13

**Authors:** Ilseob Lee, Jin Il Kim, Sehee Park, Joon-Yong Bae, Kirim Yoo, Soo-Hyeon Yun, Joo-Yeon Lee, Kisoon Kim, Chun Kang, Man-Seong Park

**Affiliations:** 1 Department of Microbiology, the Institute for Viral Diseases, College of Medicine, Korea University, Seoul 02841, Republic of Korea; 2 Division of Influenza Virus, Center for Infectious Diseases, National Institute of Health, Korea Centers for Disease Control and Prevention, Osong 28159, Republic of Korea; 3 Division of AIDS, Center for Infectious Diseases, National Institute of Health, Korea Centers for Disease Control and Prevention, Osong 28159, Republic of Korea

## Abstract

Human infection with an avian influenza virus persists. To prepare for a potential outbreak of avian influenza, we constructed a candidate vaccine virus (CVV) containing hemagglutinin (HA) and neuraminidase (NA) genes of a H5N1 virus and evaluated its antigenic stability after serial passaging in embryonated chicken eggs. The passaged CVV harbored the four amino acid mutations (R136K in PB2; E31K in PA; A172T in HA; and R80Q in M2) without changing its antigenicity, compared with the parental CVV. Notably, the passaged CVV exhibited much greater replication property both in eggs and in Madin-Darby canine kidney and Vero cells. Of the four mutations, the PA E31K showed the greatest effect on the replication property of reverse genetically-rescued viruses. In a further luciferase reporter, mini-replicon assay, the PA mutation appeared to affect the replication property by increasing viral polymerase activity. When applied to different avian influenza CVVs (H7N9 and H9N2 subtypes), the PA E31K mutation resulted in the increases of viral replication in the Vero cell again. Taken all together, our results suggest the PA E31K mutation as a single, substantial growth determinant of avian influenza CVVs and for the establishment of a high-yield avian influenza vaccine backbone.

Avian influenza A virus (AIV) has posed a pandemic threat to humans^1^,^2^,^3^. Since the first known case of H5N1 human infection in 1997, several AIV subtypes have infected humans^4^, and the infection with the two distinct AIV subtypes, H5N1 and H7N9, has provoked severe disease burden by resulting in more than 50 and 25% of human case-fatality rates, respectively^5^,^6^. Although no cases of persistent human-to-human transmission have been confirmed yet, recent reports describing aerosol transmission of the H5N1 virus in ferrets highlight the possibility of an AIV pandemic^2^,^7^,^8^.

To prepare against AIV human infection, a vaccine is considered the best medical countermeasure, and an embryonated chicken egg is a well-established platform for influenza vaccine production^9^. However, to rely solely on the eggs can be problematic^10^,^11^. One concern is that a concurrent AIV outbreak will also occur in poultry. This may cause a shortage of the eggs and the subsequent failure to provide enough substrates for vaccine production in time^12^. Another concern is the yield of a vaccine virus. In general, the internal gene backbone of A/Puerto Rico/8/34 (PR8, H1N1) virus grants efficient growth of a certain vaccine virus in the eggs. However, as observed previously^13^,^14^, the vaccine virus may not grow well in the eggs at the time of its urgent need. This often delays a vaccine manufacturing process and may increase our vulnerability to influenza. To cope with the drawbacks of the egg-based vaccine platform, an adjuvant, recombinant protein expression system, or mammalian cell-based approach has been sought by many global vaccine manufacturers^15^,^16^,^17^.

Without using adjuvants or protein expression systems, the most efficient way to prepare a large amount of vaccine may be a cell-based method^12^,^18^. This method is quicker than classical egg-based vaccine production technology and is relatively free from bacterial contamination, egg protein-related abnormalities, and egg-adapted mutations of vaccine seeds. In addition, cell-based vaccine production allows for greater flexibility in production volume and may include more cross-reactive antibodies than egg-grown vaccines^19^,^20^. Among the continuous cell lines approved by the World Health Organization for influenza vaccine, the Vero cell has been safely and successfully used for human vaccine production^21^,^22^. Recently, the first Vero cell-grown candidate vaccine virus (CVV) against a clade I H5N1 virus was licensed^22^. Most AIVs, including H5N1, grow well in Vero cells whereas human influenza viruses replicate poorly^23^. One of the reasons for this is that the higher endosomal pH of the Vero cell is well-suited to the higher fusion pH required by most AIV HA proteins^24^. However, the growth of AIVs in Vero cells is generally slower than in MDCK cells or eggs^25^, and improving the slow growth rate of AIV vaccine viruses in Vero cells is highly desirable^26^.

Here, we report the identification of a growth-enhancing mutation in the N-terminal region of the polymerase acidic (PA) protein of the PR8 influenza vaccine backbone. This PA amino acid mutation increases viral growth in the embryonated chicken eggs and vaccine cell lines for avian influenza CVVs of various subtypes. We demonstrate that the enhanced polymerase complex activity conferred by the PA amino acid mutation may underlie increased vaccine yields and HA contents for the tested CVVs. We then discuss the universal applicability of this mutation as a determinant of a high yield genetic backbone for influenza vaccine production.

## Results

### Growth properties of the H5N1 CVV and the mutations retained after serial passaging

**Figure 1:**
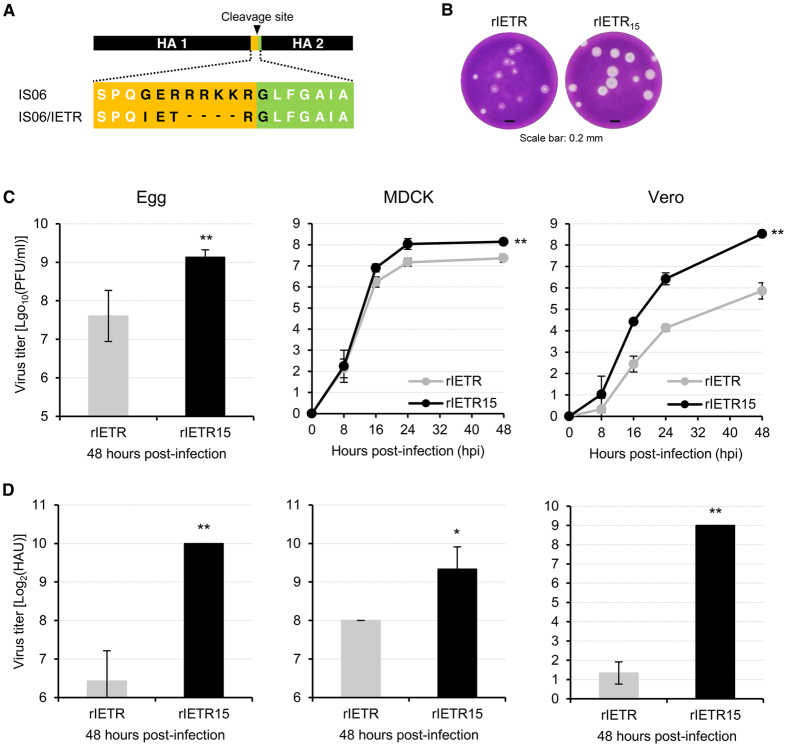
Plaque morphology and growth properties of the rIETR and rIETR_15_ CVVs. (**A**) Schematic representation of genetic modification of the HA cleavage site. (**B**) The plaque phenotypes of rIETR and rIETR_15_ CVVs. MDCK cells were infected with a similar titer of rIETR and rIETR_15_ and stained with crystal violet. Scale bar at the bottom indicates 0.2 mm. (**C**) Viral replication (**C**) and HA (**D**) titers of rIETR and rIETR_15_ CVVs were compared in the embryonated chicken eggs, MDCK, and Vero cells. After inoculation, viral replication property was determined at 8, 16, 24, and 48 hpi (for the eggs, 48 hpi only) by the plaque assay in MDCK cells. Comparison of HA contents between rIETR and rIETR_15_ was done using the cell supernatants collected at 48 hpi. The results were averaged from three independent experiments. Error bars denote standard deviation (SD). Statistical significance of viral titer differences was analyzed using Student’s t-test (*P < 0.05 and **P < 0.01).

Using the HA and NA genes of A/chicken/Korea/IS/2006 (IS06; a highly pathogenic avian influenza H5N1 virus isolated in Korea, clade 2.2), we constructed a H5N1 CVV and referred to as rIETR, based on the amino acid sequence at the modified HA cleavage site of the IS06 virus (Fig. 1A). When serially passaged 15 times in embryonated chicken eggs, the master seed rIETR and the passaged rIETR_15_ CVVs exhibited similar immunogenic properties (Table S1). However, we observed a large change in the plaque phenotypes between rIETR and rIETR_15_. As presented in Fig. 1B, rIETR_15_ produced much larger plaques than did its master seed. Consistent with the increase in plaque size, rIETR_15_ outgrew the master seed on all tested growth substrates (Fig. 1C). Along with these results, rIETR_15_ produced much increased HA titers (Fig. 1D). Notably, the increase in HA titers was largest in Vero cells.

**Figure 2:**
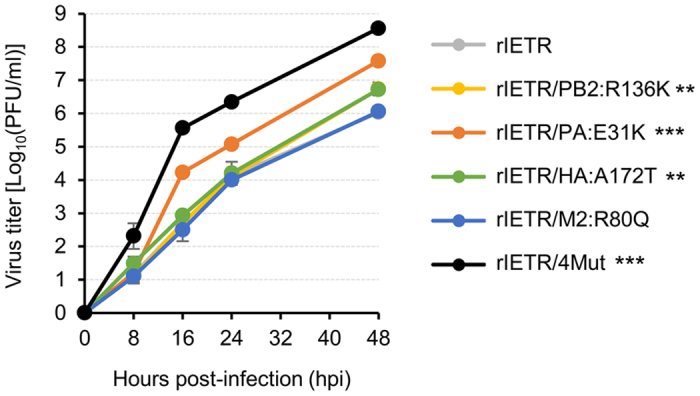
Effects of the identified mutations on the growth properties of rIETR. Replication kinetics of rIETR, rIETR/PB2:R136K, rIETR /PA:E31K, rIETR/HA:A172T, rIETR/M2:R80Q, and rIETR/4Mut viruses (please see Table 1 for the specifications) were analyzed in the Vero cells. Viral titers were determined using the cell supernatants collected at 8, 16, 24, and 48 hpi by the plaque assay in MDCK cells. The results were averaged from three independent experiments. Error bars denote SD. Statistical significance of differences was analyzed only for the viral titers determined at 48 hpi using Student’s t-test (**P < 0.01 and ***P < 0.001).

We identified four genetic mutations from rIETR_15_, which might contribute to the altered characteristics of rIETR_15_. These mutations included R136K in the PB2, E31K in the PA, A172T in the HA, and R80Q in the M2 protein (Table 1). To address how these mutations affected the growth characteristics of rIETR_15_, we generated the five mutant viruses harboring each mutation (rIETR/PB2:R136K, rIETR/PA:E31K, rIETR/HA:A172T, and rIETR/M2:R80Q) and all of the four mutations (rIETR/4Mut) (Table 1) and evaluated their replication properties (Fig. 2). For this assay, we only used the Vero cells because of its ability to support the largest increases in the viral yields and HA contents (Fig. 1C,D). As demonstrated in Fig. 1C, rIETR/4Mut, which harbored the same mutations with rIETR_15_, grew up to a 10^8.56^ plaque forming unit (PFU)/ml titer at 48 hours post-infection (hpi) whereas rIETR reached only to 10^6.03^ PFU/ml. Of the four single mutant viruses, rIETR/M2:R80Q (maximum titer, 10^6.06^ PFU/ml) exhibited a similar replication rate to rIETR, and rIETR/PB2:R136K and rIETR/HA:A172T appeared to have slightly increased replication properties. Intriguingly, rIETR/PA:E31K exhibited the highest increase of replication property among the single mutant viruses. Its replication titers always surpassed those of the other single mutant viruses at every time point (8, 16, 24, and 48 hpi) and reached up to 10^7.58^ PFU/ml at 48 hpi (Fig. 2). Even though combination of the four mutations had the highest impact, the PA E31K mutation appeared to make a major contribution to the replication of rIETR_15_ in the Vero cells. Combined, our results demonstrate that the PA E31K mutation can be a single molecular determinant to increase virus yields and HA contents of avian influenza CVVs.

### Contribution of the PA E31K mutation to viral polymerase complex activity

**Figure 3:**
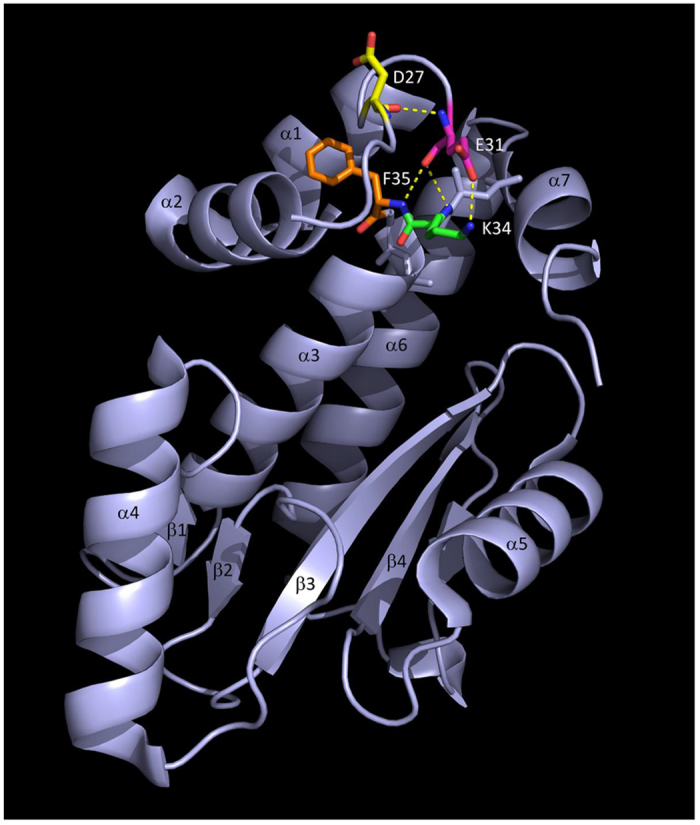
Structural representation of the molecular interactions of amino acids at PA 31 and neighboring residues. Electrostatic interaction of an amino acid signature at PA 31 to neighboring residues was investigated using a 3D structure (PDB ID: 5EGA)^46^ of PA N-terminal region of a 2009 pandemic H1N1 strain in a PyMOL software^47^. Amino acids in the target residues were indicated with different colors: aspartic acid at residue 27 (D27), yellow; glutamic acid at residue 31 (E31), magenta; lysine at residue 34 (K34), green; and phenylalanine at residue 35 (F35), orange. α1 to α7 indicate α-helices of the PA endonuclease domain, and β1 to β4 do β-sheets.

**Figure 4:**
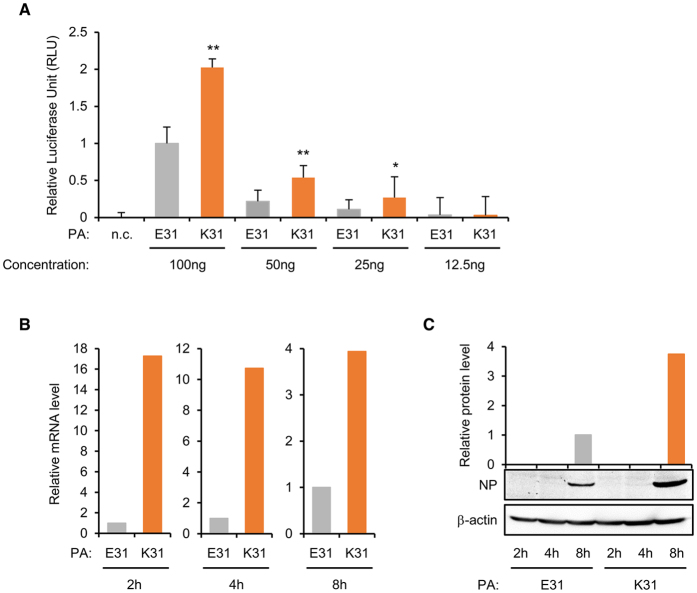
Effects of the PA E31K mutation on polymerase complex activity. (**A**) The effects of PA E31K mutation on polymerase complex activity was analyzed in the Vero cells by a Gaussia luciferase, mini-replicon assay. The Vero cells were transfected with the pDZ-PB2, PB1, PA, and NP plasmids including the pPolI-Gluc plasmid. Luciferase activity was determined at 12 h post-transfection. (**B**) qRT-PCR in the virus-infected Vero cells. The Vero cells were infected at an MOI of 0.01 with rIETR/PA:E31 or rIETR/PA:K31. Cellular RNA was isolated at 2, 4, and 8 hpi. cDNA was synthesized using Oligo(dT) primers. The expression level of NP mRNA was analyzed by qRT-PCR using NP-specific primer sets. Expression levels were normalized to GAPDH mRNA, and the relative expression levels of rPA:E31 and rPA:K31 infected cells were calculated by the ΔΔC_t_ method. (**C**) Western blot analysis. After infection with rIETR/PA:E31 or rIETR/PA:K31 in the Vero cells, the cell lysates were prepared at 2, 4, and 8 hpi. The NP protein was then detected by mouse polyclonal and anti-mouse IgG-HRP antibodies. β-actin was used as a control. The relative luciferase units were calculated from five independent experiments, and error bars denote SD. Statistical significance of the differences of luciferase expression was analyzed using Student’s t-test (*P < 0.05 and **P < 0.01).

The E31K mutation in the PA protein, which is one of the four subunits of influenza virus polymerase complex^27^, is a novel molecular alteration rIETR_15_ because almost all the PA proteins of influenza H1N1 viruses appear to possess a glutamic acid at this residue rather than lysine (Table S2). Previously, some mutations in the PA protein were reported for their association with mammalian adaptation of H5N1 viruses^28^,^29^. These mutations increased viral replication capacity in cells and pathogenicity in mice via interactions with other amino acid signatures in the same PA or other viral proteins. Likewise, we also investigated molecular interactions of amino acid signatures between the PA 31 and neighboring residues. Given the 3D structures suggested previously^30^,^31^, the residue 31 appears to be located within a loop between α2 and α3 helices of the endonuclease domain of PA protein and may interact with amino acids in the residues 27 (aspartic acid, D27), 34 (lysine, K34). and 35 (phenylalanine, F35) (Fig. 3). Based on Xiao *et al*.^31^, both glutamic acids at residues 26 (E26) and E31 may work against positively-charged K34, which ultimately hinders efficient binding between the PA endonuclease domain and viral RNA. By the PA E31K mutation, however, E31-mediated negative force disappears, and K31-mediated attractive force may catalyze efficient PA-RNA binding. Consistent with our observation, in the Vero cells transfected with the four plasmids of viral polymerase complex (PB2, PB1, PA, and NP) and a luciferase reporter plasmid, the PA E31K mutation increased luciferase expression in a dose-dependent manner (Fig. 4A). In the subsequent results of quantitative real-time PCR (qRT-PCR) and western blotting assay in the Vero cells, the PA E31K mutation also consistently increased mRNA and protein expression levels of viral NP protein, respectively (Fig. 4B and C), without showing a pathogenicity increase in mice (Table S3). These results confirm the effects of the PA E31K mutation on viral replication capacity presented in Figs 1 and 2. Considered together, our results indicate that the PA E31K mutation may confer replication enhancement to influenza vaccine viruses by increasing viral polymerase activity through an efficient interaction between PA protein and viral RNA, which may, in turn, contribute to the higher expression of viral RNAs and proteins.

### Application of PA E31K to other avian CVVs

**Figure 5:**
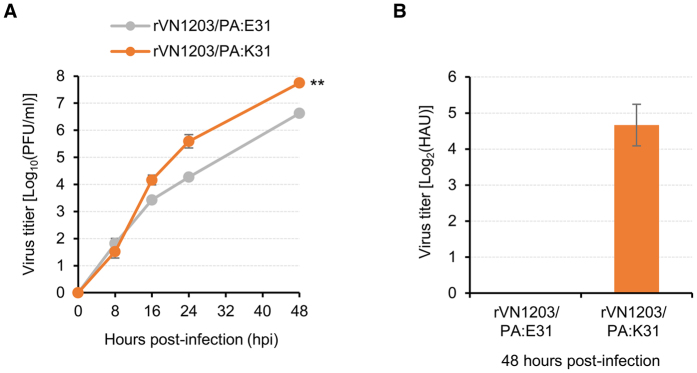
Effect of the PA E31K mutation on the growth property of a clade 1 H5N1 CVV. The Vero cells were inoculated with rVN1203/PA:E31 or rVN1203/PA:K31 at an MOI of 0.01. Viral titers were determined using the cell supernatants collected at 8, 16, 24, and 48 hpi by the plaque assay in MDCK cells (**A**). The HA contents were determined only using the 48 hpi cell supernants (**B**). The average viral titer or geometric mean HA titers were calculated from three independent experiments, and error bars denote SD. Statistical significance of viral titer differences was analyzed using Student’s t-test (**P < 0.01).

We observed the beneficial effects of the PA E31K mutation on the viral replication property of the H5N1 CVV (Figs 1 and 2). To further evaluate the feasibility of the PA mutation as a high yield determinant for other CVVs, we generated two additional H5N1 CVVs using the HA and NA genes of A/Viet Nam/1203/2004 (VN1203, clade 1). The new H5N1 CVVs rVN1203/PA:E31 and rVN1203/PA:K31 retained the PA E31 and K31, respectively. In the replication kinetics analysis in the Vero cells, rVN1203/PA:K31 grew up to 10^7.8^ PFU/ml whereas rVN1203/PA:E31 reached only to 10^6.6^ PFU/ml (Fig. 5A). More drastic differences were observed with their HA titers. The HA titer of rVN1203/PA:E31 was barely detectable in three different experiments (Fig. 5B). However, as its higher yield of replication in the Vero cells (Fig. 5A), rVN1203/PA:K31 resulted in a 2^4.6^ HA titer (Fig. 5B).

**Figure 6:**
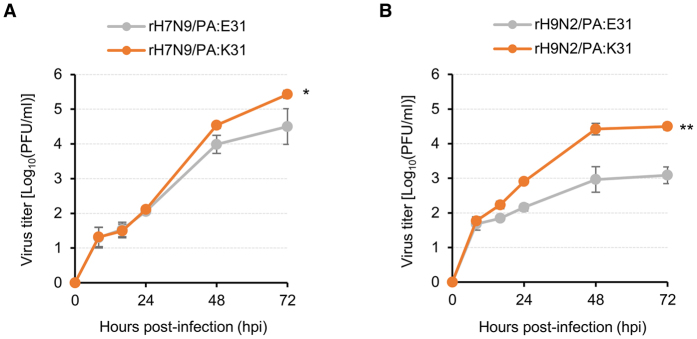
Effect of the PA E31K mutation on the growth properties of H7N9 and H9N2 CVVs. The Vero cells were inoculated with (**A**) H7N9 (rH7N9/PA:E31 or rH7N9/PA:K31) and (**B**) H9N2 (rH9N2/PA:E31 or rH9N2/PA:K31) CVVs at an MOI of 0.01. Viral titers were determined using the cell supernatants collected at 8, 16, 24, 48, and 72 hpi by the plaque assay in MDCK cells. Viral titers were averaged from three independent experiments, and error bars denote SD. Statistical significance of viral titer differences was analyzed using Student’s t-test (*P < 0.05 and **P < 0.01).

The effects of the PA E31K mutation on viral replication in the Vero cells were also evaluated on the other subtype CVVs. The H7N9 (A/Anhui/01/2013) and H9N2 (A/Chicken/Korea/) CVVs were generated by harboring either PA E31 or PA K31. The H7N9 and H9N2 CVVs exhibited reduced growth rates in the Vero cells, compared with the H5N1 CVVs. However, rH7N9/PA:K31 and rH9N2/PA:K31 resulted in the higher growth rates, compared with rH7N9/PA:E31 and rH9N2/PA:E31, respectively (Fig. 6A and B). Even though the HA titers of the H7N9 and H9N2 CVVs were below the detection limit (data not shown), the PA E31K mutation demonstrated its beneficial effects on replication properties of H7N9 and H9N2 subtype CVVs. Considered all together, our results suggest the feasibility of the PA E31K mutation as a high yield, molecular determinant of avian influenza CVVs.

## Discussion

During the evaluation process of antigenic stability of the avian influenza H5N1 CVV by the 15 times, serial passages in embryonated chicken eggs, we observed enlarged plaques of the passaged rIETR_15_, compared with those of the master seed rIETR (Fig. 1B). It was then revealed that the enlarged plaques of rIETR_15_ correlated with its increases in viral growth yields and HA contents in the chicken eggs, MDCK cells, and Vero cells. Given the importance of high yield characteristics of CVVs in terms of vaccine manufacturing, our results may underline a serial adaptation process in certain substrates as a way of preparing an influenza vaccine virus or vaccine backbone. To narrow down what molecular changes brought in the increases of plaque size and viral replication, we compared the genomic sequences of the master seed rIETR and the passaged rIETR_15_ CVVs and identified the four amino acid mutations from rIETR_15_:PB2 R136K, PA E31K, HA A172T, and M2 R80Q (Table 1). Each mutation was used to generate the four single mutant viruses. Individual effects of these mutations on the replication property was then evaluated in the Vero cells. Compared with rIETR, the four single mutant viruses (rIETR/PB2:R136K, rIETR/PA:E31K, rIETR/HA:A172T, and rIETR/M2:R80Q) exhibited similar or increased replication properties, and of them, rIETR/PA:E31K resulted in the most enhanced replication titer (Fig. 2). Even though rIETR/4Mut, which harbored all the four mutations, showed the most increased replication property, we tried to find a single molecular determinant of high yield influenza CVVs and focused on the PA E31K mutation based on its beneficial effects on viral replication in the Vero cells.

The PA protein is one of the four proteins that consist of the polymerase complex of influenza viruses^27^. Several reports have described the replication benefits resulted from a single amino acid mutation in one of the four polymerase complex proteins^32^,^33^,^34^. Even though another study indicated no correlation between viral replication and polymerase activity^35^, there may be some contradictions between different virus subtypes and strains. We also examined whether the PA E31K mutation would increase viral polymerase complex activity. As suggested by the structural analysis (Fig. 3), the PA E31K mutation surely increased luciferase expression in a dose-dependent manner, which was then confirmed by RNA and protein expression levels (Fig. 4). These all results suggest that a single amino acid mutation in the PA protein, not multiple mutations in one or more viral proteins^28^,^36^,^37^, is enough to increase viral yields and HA contents of avian influenza CVVs without affecting viral pathogenicity (Table S3).

We also evaluated the beneficial effects of the PA E31K mutation on viral replication of the other H5N1, H7N9, and H9N2 CVVs (Figs 5 and 6). Interestingly, PA E31K-mediated growth enhancement was bigger in the Vero cells than in the MDCK or in the chicken eggs, even though the PA E31K mutation was arisen during a viral adaptation process in the eggs. The Vero cells are considered to have an impaired type I interferon response^38^ whereas that of MDCK cells is only partially impaired^39^. Hence, the loss of interferon-mediated viral defense mechanisms might create a permissive environment for our avian CVVs. However, the beneficial effects of the PA E31K mutation on viral replication in the Vero cells could be more dependent of pH condition for HA fusion activity than defective type I interferon response because most human influenza viruses do not grow well in the Vero cells, compared with avian viruses^24^,^40^. Extension of the PA E31K mutation to human influenza CVVs should be further exploited for the development of diverse cell culture-based influenza CVVs in future studies.

The N-linked glycosylation (NLG) of the HA protein is considered to affect the antigenicity, immunogenicity, pathogenicity, and transmission of influenza viruses^41^,^42^,^43^. Changing an amino acid signature at HA residue 172 (which corresponds to HA position 160 under H3 numbering) might generate a potential NLG site at HA residue 170. The NLG status of this residue appears to be important for the aerosol transmission of a H5N1 virus between ferrets^7^,^8^. In addition, the presence or absence of NLG at this residue can affect viral antigenicity and pathogenicity, in conjunction with nearby NLG sites^44^. Consistent with a previous observation^44^, we obtained an approximately 10-fold increase of viral replication following this HA mutation (Fig. 2). However, we did not evaluate the effect of this HA mutation in cooperation with other mutations on the viral yields and immunogenicity of avian influenza CVVs. These combinations should be also investigated in course of establishing an efficient vaccine backbone for the influenza viruses.

In conclusion, we demonstrated that the PA E31K mutation could increase viral yields and HA contents of different subtypes of avian influenza CVVs. By increasing the activity of viral polymerase complex, this mutation enhanced viral replication properties of the avian influenza CVVs in the Vero cells. Based on these, we suggest the PA E31K mutation as a feasible growth determinant of avian influenza CVVs.

## Methods

### Ethics

This study was conducted in strict accordance with the recommendations in the Guide for the Care and Use of Laboratory Animals of the Animal, Plant, and Fisheries Quarantine and Inspection Agency of Korea. The protocols for mouse and embryonated chicken egg experiments were approved by the Institutional Animal Care and Use Committee of Korea University College of Medicine (permit number: KUIACUC-2014-249).

### Viruses, plasmids, and cells

Avian influenza A(H9N2) A/chicken/Korea/01310/2001 virus was provided by Dr. Young Ki Choi (Chungbuk National University, Cheongju, Republic of Korea). The HA (GISAID EpiFlu database: EPI439507) and NA (EPI439509) genes of a human-infecting influenza A(H7N9) A/Anhui/01/2013 virus were synthesized by Cosmogenetech (Seoul, Republic of Korea). The HA and NA genes of a highly pathogenic avian influenza A(H5N1) A/Chicken/Korea/IS/2006 (IS06, clade 2.2.) virus were provided by the Korea Centers for Disease Control and Prevention (Osong, Republic of Korea). For the IS06 HA, polybasic amino acids (-RRRKKR/G-) at the cleavage site were modified into a single basic amino acid (-R/G-) before its use (please see Fig. 1A)^10^. All the HA and NA genes were cloned into a bidirectional pDZ plasmid for reverse genetics. The pDZ plasmids of HA and NA genes of a highly pathogenic avian influenza A(H5N1) A/Vietnam/1203/2004 (VN1203, clade 1) virus and six internal genes (PB2, PB1, PA, NP, M, and NS) of a human vaccine donor A/Puerto Rico/8/34 (H1N1) virus were provided by Dr. Peter Palese (Icahn School of Medicine at Mount Sinai, New York, NY). All H5N1-related experiments were performed at a BSL3 facility at the Korea Center for Disease Control and Prevention. MDCK, 293 T, and Vero cells were purchased from ATCC (Manassas, VA). MDCK cells were maintained in Eagle’s minimum essential medium (Lonza, Basel, Switzerland), and 293 T and Vero cells were maintained in Dulbecco’s modified eagle’s medium (Gibco, Waltham, MA). Each cell medium was supplemented with 10% FBS, 1% penicillin/streptomycin, and the cells were maintained at 37 °C, 5% CO_2_ incubator.

### Generation of avian influenza CVVs

Avian influenza CVVs and mutant viruses were generated by plasmid-based reverse genetics as described previously^42^,^45^. Briefly, eight plasmids encoding each viral RNA and protein were transfected into 293T-MDCK co-cultured cells and supplemented with 1 μg/ml of TPCK-treated trypsin. After 48–72 hours, cell supernatants were harvested and inoculated into 10-day-old embryonated chicken eggs to propagate the recombinant virus. The allantoic fluids were collected at 48 hours post-infection (hpi), and the presence of virus was examined by a hemagglutination (HA) assay. All recombinant viruses were plaque-purified in MDCK cells and confirmed by sequence analysis before use.

### Serial passaging of CVV in embryonated chicken eggs

After primary propagation, the rIETR CVV was serially passaged in 10 days old embryonated chicken eggs. For every passage, 10^3^ PFU of viral allantoic fluid was inoculated into the eggs. Each passaged virus was collected from allantoic fluid at 48 hpi and titrated by a plaque assay in MDCK cells. After the 15^th^ passage, the rIETR_15_ CVV was selected among the egg-adapted variants by purification in MDCK cells, based on the size of plaques, and used for the subsequent analyses.

### Viral growth kinetics and HA contents

The replication kinetics of the viruses were analyzed in MDCK and Vero cells. Briefly, the monolayer cells were infected with viral inoculum (multiplicity of infection, MOI = 0.01) for one hour, washed five times with cell culture medium, and maintained at 37 °C, 5% CO_2_ with a new medium containing 1 μg/ml TPCK-trypsin. Cell supernatants were collected at 8, 16, 24, and 48 hpi for the titration by the plaque assay in MDCK cells. To evaluate viral growths in the embryonated chicken eggs, 10^3^ PFU virus was inoculated into the allantoic cavity. At 48 hpi, allantoic fluids were collected and titrated by the plaque assay. HA titers of the viruses were determined by a hemagglutination (HA) assay using turkey red blood cells (tRBCs). Briefly, 50 μl of allantoic fluid was serially, two-fold diluted in PBS. Then, 50 μl of a 0.5% (volume/volume) tRBC was added to each allantoic fluid. After 30–45 minutes of incubation at 4 °C, HA titers were calculated as the reciprocal of the highest dilution with complete HA activity.

### Plaque assay

A standard plaque assay was performed to determine the actual infectious viral titer. Briefly, MDCK cells were inoculated with viral allantoic fluids for one hour. After discarding the inoculum, the cells were washed three times with PBS and overlaid with a medium containing 0.2% agar, 1 μg/ml trypsin and maintained at 37 °C, 5% CO_2_. The cells were then stained with crystal violet at 72 hpi, and viral titers were determined by counting the number of plaques.

### Mini-replicon assay and quantitative real-time PCR

Viral polymerase activity was analyzed in Vero cells with a Gaussia luciferase mini-replicon assay. The Gaussia luciferase gene was inserted as a reporter between PR8 NA non-coding regions (pPolI-Gluc plasmid). The Vero cells at 70–80% confluency were transfected with the four plasmids expressing viral polymerase complex proteins (PB2, PB1, PA, and NP) and pPolI-Gluc plasmid using TransIT-2020 (Mirus Bio LLC, Madison, WI). At 12 hours post-transfection, luciferase expression was estimated and adjusted by WST-1 treatment (Roche, Germany). For quantitative real-time PCR (qRT-PCR), the Vero cells were inoculated at an MOI of 0.01 with rIETR/PA:E31 or rIETR/PA:K31. The cellular RNA was isolated at the indicated time point, and cDNA was synthesized using Oligo(dT) primer. The expression of NP mRNA was analyzed using NP-specific primer sets. The RNA expression was then normalized using that of GAPDH mRNA, and the relative expression levels in rIETR/PA:E31- and rIETR/PA:K31-infected cells were calculated by the ΔΔC_t_ method.

### Western blot analysis

Vero cells were infected at an MOI of 3. At 2, 4, and 8 hpi, the cell lysates were treated with RIPA buffer (Life Technologies, Carlsbad, CA) and prepared for SDS-PAGE analysis. NP in the cell lysates was primarily detected by mouse anti-IS06 polyclonal antibodies and then secondarily detected by anti-mouse IgG-HRP antibody (KPL, Washington, DC). β-actin (Sigma-Aldrich, St. Louis, MO) was used as a control.

### Mouse experiment

Pathogenicity of rIETR/PA:E31 and rIETR/PA:K31 was analyzed in mice (BALB/c, female, 5 weeks old). Three mice per group were infected intranasally with 10^5^ PFU. At 3 and 5 days post-infection (dpi), mouse lungs were collected and homogenized for the titration of viral replication by the plaque assay. Immunogenicity of rIETR and rIETR_15_ was also analyzed in mice. Five mice per group were primed and boosted at two weeks interval with inactivated rIETR and rIETR_15_ (intramuscular injection, 128 HA units) and bled for serum collection. The serum samples were then treated with receptor destroying enzyme (neuraminidase from *Vibrio cholerae*; Denka Seiken, Japan) and used for a hemagglutination inhibition (HI) assay. Briefly, each serum sample was two-fold diluted in PBS and incubated with four HA units of viruses at 37 °C for one hour. After incubation, 50 μl of 0.5% (volume/volume) tRBCs were added into each well, and HI titers were determined after another 45 minutes.

## Additional Information

**How to cite this article**: Lee, I. *et al*. Single PA mutation as a high yield determinant of avian influenza vaccines. *Sci. Rep.* **7**, 40675; doi: 10.1038/srep40675 (2017).

**Publisher's note:** Springer Nature remains neutral with regard to jurisdictional claims in published maps and institutional affiliations.

## Supplementary Material

Supplementary Information

## Figures and Tables

**Table 1:** Amino acid mutations identified from the rIETR_15_ CVV and individual mutant viruses.

		Amino acid signature in the protein position^‡^			
		PB2	PA	HA	M2
Classification	Virus	136	31	172	80
Wild-type	rIETR	R	E	A	R
Passaged	rIETR_15_	R/K	K	T	Q
^†^rg-Mutant	rIETR/4Mut	K	K	T	Q
	rIETR/PB2:R136K	K	—	—	—
	rIETR/PA:E31K	—	K	—	—
	rIETR/HA:A172T	—	—	T	—
	rIETR/M2:R80Q	—	—	—	Q

^†^Reverse genetics;

^‡^Amino acid signatures at each residue were identified from at least three different plasmid clones.
