# Supplementary material for: Single PA mutation as a high yield determinant of avian influenza vaccines
Source: Sci Rep. 2017 Jan 13;7:40675. doi: 10.1038/srep40675 (PMC5233958; doi:10.1038/srep40675)
Supplement: Supplementary Information [file srep40675-s1.pdf]

Supplementary information

Single PA mutation as a high yield determinant of avian influenza vaccines

Authors: Ilseob Lee, Jin Il Kim, Sehee Park, Joon-Yong Bae, Kirim Yoo, Soo-Hyeon Yun, Joo-Yeon Lee, Kisoon Kim, Chun Kang, Man-Seong Park

Table S1 Page 3

Table S2 Page 4

Table S3 Page 5

Supplementary tables and footnotes

Table S1. Serum HI titers determined before and after immunization in mice.

	Serum GMT (95% CI§)		
	Before immunization	After immunization	
Virus	Homologous	α-rIETR	α-rIETR$_{15}$
rIETR	0	31.75 (11.75-85.80)	15.87 (5.87-42.90)
rIETR$_{15}$	0	31.75 (11.75-85.80)	63.50 (23.49-171.6)

§, Confidence interval.

Table S2. Frequency of amino acid mutations at the respective residues of influenza A PB2, PA, HA, and M2 protein genes.

Mutation	The number of sequences	Occurrence (%)	Other amino acid signatures
PB2 R136K	6,831 (H1N1)	0	R136 (6,685; 97.86%), others (146; 2.14%)
PA E31K	6,575 (H1N1)	0	E31 (6,574; 99.99%), V31 (1; 0.01%)
HA A172T	3,776 (H5)	28.28	A172 (2,528; 66.95%), others (180; 4.77%)
M2 R80Q	2,194 (H1N1)	99.13	R80 (5; 0.23%), others (14; 0.64%)

Table S3. Viral titers in the lungs of infected mice.

Virus	Virus titer [$\log_{10}$(PFU/ml/g)] ± SD[§]	
	3 dpi	5 dpi
rIETR	5.93 ± 0.08	5.76 ± 0.41
rIETR/PA:E31K	5.76 ± 0.03	5.44 ± 0.26

[§], Standard deviation.
